# Research imperative

**DOI:** 10.1016/j.conctc.2019.100350

**Published:** 2019-03-24

**Authors:** Howard Trachtman

**Affiliations:** Department of Pediatrics, Division of Nephrology, NYU, Langone Health, New York, NY, 10016, USA

## Abstract

There is a note of caution expressed when clinical care providers enroll their own patients into investigational trials, a concern expressed in the called dual-role consent. There is concern that this circumstance may create a conflict of interest for the physician-investigator, lead to loss of patient voluntarism, and promote the therapeutic misconceptions. In this opinion paper, I review the circumstances surrounding participation in clinical research and the conduct of standard patient care. I propose that when a patient is eligible for an institutional review board-approved clinical trial, instead of representing a potential ethical lapse, soliciting enrollment by the clinician-researcher may represent optimal care for the patient.

## Introduction

There is an unspoken but powerful assumption in medicine that clinical researchers and primary care providers are fundamentally different from one another. They are both MDs but they engage with patients in disparate ways. To preserve this distinction, it has been suggested that a wall of separation should be built and maintained between researchers and practitioners. This attitude is reified by the cautionary stance towards clinical researchers who try to enroll patients into ongoing studies, observational or interventional. The warning has been greatest for therapeutic studies and especially when the primary care physician is the principal investigator of the trial.

Underlying the note of caution against clinical researchers obtaining consent and enrolling their own patients into clinical trials, so called dual-role consent, lurk three potential dangers: conflict of interest for the physician-investigator, loss of patient voluntarism, and promotion of the therapeutic misconceptions [1]. The implication is that advocacy for clinical research is at odds with the physician's fiduciary responsibility to his/her individual patient. Encouraging participation in trials and viewing the patient as part of a larger disease cohort is seen as being in opposition to the principle that the only consideration for the caring physician is the patient standing in front of him or her. This line of thinking has even been extended to nurses who are engaged in clinical research [2].

I suggest that this represents a mischaracterization of clinical research. I am not advocating for complete merger of clinical care and clinical research. They involve different skill sets and appeal to different types of doctors. However, in the course of managing individual patients, these two care paths can converge and under those circumstances, clinical research and patient care share a common goal, namely, identifying the best treatment for the patient. Therefore, it is ethically justified for the same physician to be involved, in fact take the lead, in patient management and enrollment into clinical trials.

Now to the details. Ethically sound interventional clinical research addresses a relevant medical problem for which there is equipoise between alternative treatment options. This means that there is genuine uncertainty about the best treatment option for a defined medical problem. Communicating what equipoise means is difficult, a reflection of physician attitudes and biases [3]. Regardless, approval of a clinical research project by an authorized institutional review board (IRB) indicates that the subject matter is important, equipoise applies, the protocol is scientifically sound, and the risks and benefits to prospective participants are in appropriate balance so that patient safety and well-being are preserved to the fullest extent possible. A clinical investigator should not rely blindly on the IRB approval and should confirm for him/herself that equipoise applies and that the risk:benefit ratio is acceptable for each potential study participant. Nonetheless, the rigorous IRB review and approval status should encourage routine consideration of trials and offers of enrollment to patients.

Therefore, if a physician is being honest about the state of medical knowledge for a given patient at a specific point in their clinical course, then a clinical trial that is germane to the patient's illness is the optimal treatment at that time. This circumstance may arise in the initial approach to therapy for rare, life threatening diseases or after sequential applications of approved therapies for more common, relatively benign health problems. There is genuine uncertainty about the efficacy of the current standard of care. The therapeutic decision under study may center on the superiority of one of two currently approved therapeutic modalities or a comparison between the standard of care and an experimental intervention. The principal investigator of the study has proposed a change in the usual treatment regimen or the adoption of a novel intervention in an attempt to increase survival, achieve better preservation of organ function, reduce toxicity, or improve patient-reported clinical outcomes. Failure to inform patients about relevant clinical trials and offering them the opportunity to enroll misrepresents the state of knowledge about current treatment choices and falls short of fully informing patients and parents of all of their therapeutic options. Under these circumstances, the best treatment approach is to promote participation in a clinical trial in an attempt to reduce the therapeutic uncertainty in the gray zones of care. Instead of compromising care, incorporating clinical research into routine care promotes an unbiased assessment and potential improvement of treatment for patients who are eligible for enrollment in a clinical study. Clinical research should be seen as an imperative for care providers to prevent therapeutic misconceptions surrounding the accepted standard of care. Clinical trials often yield negative findings. However, in an ethically sound protocol, the outcomes of the test therapy should be no worse than the standard care and, barring unanticipated events, the safety profile is considered acceptable. An investigator should confirm this determination by reviewing relevant pre-clinical data, and prior experience with the test agent or similar drugs. In addition, he/she should be fully aware of the potential participant's health status and unique risks of untoward reactions to the study drug. With all that said, one can infer that not only is it reasonable to present all relevant IRB-approved clinical trial options to a patient, it could be considered an ethical obligation in order to promote continuing efforts to define the best treatment for that patient.

There are unique situations such as pediatric oncology in which this goal has been actualized to a large degree. There are hardly any children or adolescents with a malignant condition in the United States who are not routinely offered the opportunity to enroll in the full range of clinical studies that pertain to their condition. The incorporation of relevant trials into patient management has resulted in a steady improvement in outcomes for children with a wide range of pediatric malignancies [4].

It is worth noting that the individual patient-physician relationship is subject to the same ethical threats that impact on the clinical researcher. Doctors’ recommendations to their patients are subject to influence by a range of external factors. Financial and administrative considerations such as continuation of follow-up and maintenance of a favorable institutional profile may impact on the conversation between physicians and patients. Patients may feel pressured to accept therapeutic recommendations out of concern that the doctor will be upset with a refusal. Physicians may overstate the potential benefits or underestimate adverse consequences of established treatments. Although these concerns apply in clinical trials, requirements for full disclosure to minimize investigator conflict of interest exist and are being steadily intensified to limit research misconduct. In addition, one cannot be enrolled in a clinical trial unless one satisfies the inclusion criteria and does not have any reason for exclusion. In that regard, there is greater oversight of the appropriateness of treatment in clinical trials than in routine practice. Clinical investigators are mandated to form a data monitoring committee for oversight of trial outcomes, both good and bad, a feature that is not part of one-on-one patient care. Finally, it is worth noting that participation in clinical studies may actually be associated with better outcomes for all participants. This “trial effect”, which may be attributable to improved delivery of care, better adherence to treatment, and more comprehensive clinical monitoring, applies to those assigned to the experimental treatment or placebo [5,6].

Many studies have documented the low rate that clinical trial options are offered to patients by their physicians [7]. This is especially relevant in nephrology, a discipline characterized by the lowest rate of performance of randomized clinical trials compared to all other medical subspecialties [8,9]. There is a high degree of acceptance of clinical trials by patients when opportunities are presented to them, especially when the offer is made by their doctor [10]. While broad community acceptance of clinical trials would make it easier for primary care providers to engage in this work, patients’ choices should always be respected. They are not obligated to participate in clinical trials and always retain the right to refuse or withdraw from trials. Providing the standard of care for those who opt for it is always acceptable for patients and families who may be unable to tolerate the uncertainties raised by enrolling in clinical trials. However, educating physicians and patients that the performance of clinical research is a valid and integral aspect of overall care and encouraging enrollment of patients by their physicians into suitable trials should be considered optimal management rather than a betrayal of the individual doctor-patient relationship.
